# Efficiency of pivot splints as jaw exercise apparatus in combination with stabilization splints in anterior disc displacement without reduction: a retrospective study

**DOI:** 10.1186/1746-160X-10-42

**Published:** 2014-10-09

**Authors:** Mehmet Muhtarogullari, Mehmet Avci, Bulem Yuzugullu

**Affiliations:** Department of Prosthodontics, Faculty of Dentistry, Hacettepe University, Ankara, Turkey; Private practice, Istanbul, Turkey; Department of Prosthodontics, Faculty of Dentistry, Baskent University, Ankara, Turkey

**Keywords:** Temporomandibular joint, Internal derangement, Anterior disc displacement without reduction, Magnetic resonance imaging, Pivot splint, Stabilization splint

## Abstract

**Objective:**

To evaluate efficiency of pivot splints in jaw exercises, in combination with stabilization splints, in cases of anterior disc displacement without reduction of temporomandibular joint.

**Subjects and methods:**

Twenty-three patients who referred to the prosthodontics clinic in 1995–1997 were included in the study, where anterior disc displacement without reduction of temporomandibular joint was diagnosed using magnetic resonance imaging and clinical examination. Pivot splints were used for jaw exercises for five minutes long; five times/day and stabilization splints were used at all other times. The patients were followed for 24 weeks. Lateral and protrusive excursions along with maximum mouth opening and were evaluated at each control. Bilateral palpation of temporal, masseter, sternocleidomastoid muscles and TMJ was assessed for pain perception before and after treatment. Data were statistically analyzed using Paired sample t-test and Independent Samples t-test (p < .05).

**Results:**

Mean mandibular range of motion measurements increased from 28.74 mm prior to 49.17 mm on maximum opening; right/left lateral excursion from 7.61 mm to 12.04 mm and 4.09 mm to 7.3 mm on protrusion after treatment. All changes observed before and after treatment were found to be statistically significant. (p < .001) Pain symptoms were eliminated at the end of 24 weeks of treatment in all patients.

**Conclusion:**

Using pivot splints as an exercise regimen along with a stabilization splint may be a viable treatment option for patients with anterior disc displacement without reduction; as normal mandibular range of motion was established and pain was eliminated.

## Introduction

Anterior disc displacement without reduction (ADDWoR) of the temporomandibular joint (TMJ), ‘closed lock’ is a widespread disorder that clinically presents itself with restriction in jaw movements, in which the morphology of the disc is altered while the discal ligaments have become elongated [1, 2]. The longer the disc is displaced anteriorly and medially, the greater the thinning of its posterior border and the more the lateral discal ligament and inferior retrodiscal lamina will be elongated. Also, protracted anterior displacement of the disc will lead to a greater loss of elasticity in the superior retrodiscal lamina. The disc can be forced through the discal space, eventually collapsing the joint space behind it, trapping the disc in the forward position [3]. During mouth opening the effected joint exhibits rotation, but translation is limited or non-existent [4, 5]. In these circumstances, providing function by re-establishing the ideal disc-condyle relationship or more often by reducing restriction in movement should be the goal of the treatment to eliminate pain [5]. When patients complain about being locked for a week or less, manipulation to recapture the disc could be attempted. However, if recapturing cannot be accomplished, different approaches such as splint therapies, arthroscopic and/or open joint surgeries might be considered to reduce functional limitations along with pain control [3, 6, 7]. Nonsurgical therapy should be the first treatment choice to prevent risk of postoperative surgical complications although there were instances where surgical interventions may be successful. The splints may be classified into three major groups with respect to their hypothesized function: relaxation/stabilization splints, distraction/pivot splints and repositioning splints. The latter have been described for the therapy of painful disc displacement with reduction [8].

The purpose of this retrospective study was to investigate the efficiency of a treatment approach that consists of using pivot splints for jaw exercises in combination with stabilization splints in cases of ADDwoR with former unsuccessful manual reduction attempts history.

## Methods

### Study population

Twenty-three patients (3 male, 20 female) in an age range of 24 and 48 (mean age 27.1), referred to the Department of Prosthodontics in Hacettepe University, Faculty of Dentistry with the chief complaint of symptoms in the temporomandibular joint (TMJ) between the years of 1995–1997 were screened from the archives. The study was approved by the Ethics Committee of Hacettepe University (GO 14/97).

The inclusion criteria were: patients over 18 years old; previous history of limited mouth opening for more than 2 weeks; pain in the TMJ area aggravated by jaw movement and function; a positive diagnosis of unilateral or bilateral ADDwoR by means of magnetic resonance imaging (MRI); maximum mouth opening of <40 mm; and previous attempts of unsuccessful manual reduction. Patients who were unwilling or unable to receive splint and/or exercise therapy; had previously been treated for temporomandibular joint (TMJ) disorders (TMD); had extensive restorations, missing teeth, fixed or removable partial dentures; had systemic rheumatic disease, generalized joint pain or swelling, neurologic disorders, had concurrent use of steroids, anti-inflammatories, muscle relaxants or narcotics, major psychiatric disease and prior TMJ surgery were excluded.

The same operator performed all clinical examination, splint therapy and control in the follow-up appointments of all patients.

### Baseline measurements

#### Mouth opening

The patients were asked to open their mouth as wide as possible to measure the incisal edge clearance in millimeters (mm), The distance between the first right incisor of the maxilla and mandibula was measured with a millimetric ruler.

#### Lateral excursions

The patients were asked to open their mouth slightly (physiological rest position) and move their mandible as far as possible towards right or left (maximum lateral position). The midline labioincisal embrasure of the mandibular incisor was measured with a millimetric ruler.

#### Protrusion

The initial position was the physiological rest position from which the patient moved the mandible anteriorly without contacting the teeth. The distance from the incisal edge of the maxillary central incisor to the incisor edge of the mandibular incisor was measured in the maximum protruded position.

#### Pain assessment

The temporal, masseter, sternocleidomastoid muscles and TMJ were palpated bilaterally for pain perception.

### Magnetic resonance imaging

Magnetic resonance imaging had been performed at Hacettepe University, Faculty of Medicine, Department of Radiology, with 0,5 Tesla MR scanner (Gyroscan, Phillips, Netherland) prior to treatment. T1 weighted oblique-sagittal images of both joints were obtained in 3 mm thick slices for each patient by using surface coil attachments with an internal diameter of 11 cm and an external diameter of 14 cm. Sequential bilateral images were obtained of the closed mouth and the maximal open mouth positions. Normal disc position was defined as the posterior band of the disk located superior of the head of the mandibular condyle. Disk displacement was defined as having the posterior band of the disk located anterior to the mandibular condyle.

### Treatment protocol

Each patient received two maxillary splints made of clear auto polymerizing acrylic resin. Pivot splints were fabricated from acrylic resin and adjusted intraorally as described by Sears [9], with a bilateral pivot in the region of the second molar teeth.

Full arch maxillary stabilization splints were adjusted to have uniform and simultaneous contacts with the buccal cusp tips of posterior and incisor edges of the anterior teeth of the opposing arch. Eccentric guidance was established with acrylic prominences labial to the mandibular canines to have disclusion in the posterior teeth.

**Figure 1:**
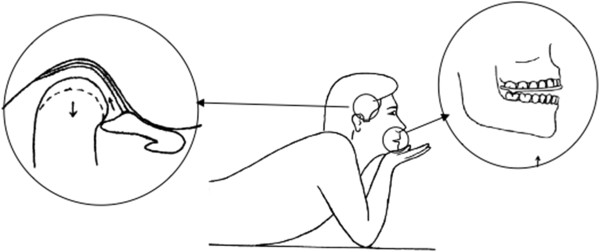
**Exercise position with the pivot splint.**

An exercise regimen of five minutes long, five times/day, with a minimum of three hours between each exercise, was recommended. In the exercise period, patients were asked to lie on a hard flat surface and exert force under the chin with one hand in an upward direction with the pivot splint in place (Figure 1). The patients were only allowed to remove the stabilization splints during mealtimes, oral hygiene procedures and daily exercises with the pivot splint. No muscle relaxants, analgesics or anti-inflammatory agents were prescribed during the course of the treatment. Clinical examinations were performed on a weekly basis for 24 weeks.

At all evaluation days, all patients were extensively informed about that overuse, misuse or parafunction could enhance or provoke their complaints. They received instructions to keep the jaw muscles relaxed, and avoid non-functional tooth contacts and excessive mouth opening.

During each evaluation appointment; maximum interincisal opening, protrusive and lateral excursions were recorded as assessed for the baseline measurements, TMJ, sternocleidomastoid, masseter, temporal and lateral pterygoid muscles were palpated and stabilization splints were adjusted if necessary. Treatment procedures were continued for 24 weeks. Magnetic resonance imaging had been performed at the end of the treatment period as described previously.

### Statistical analysis

Paired sample t-test was used to determine the changes in the range of motion before and after treatment. Mean values of the range of motion (mm) of patient groups with change or no change in disc positions after treatment; related to age, locking duration, maximum mouth opening, lateral and protrusive eccentric movements were compared using the Independent Samples t-test.

## Results

The mean average time from the onset of limited mouth opening was 13,74 ± 9,99 weeks. Mean mandibular range of movement measurements are shown on Table 1. All changes observed before and after treatment were found to be statistically significant (p < .001) (Table 1).

**Table 1:** **Changes in the range of movements (mm) before and after treatment**

Movements	Before treatment*	After treatment*	t	p-value
Right/Left lateral movement	7,61 ± 1,69	12,04 ± 1,41	12,990	<0.001
Protrusive movement	4,09 ± 1,41	7,3 ± 1,43	7,990	<0.001
Maximum interincisal opening	28,74 ± 5,51	49,17 ± 6,37	14,470	<0.001

*Results are expressed as mean ± standard deviation.

Differences in mean values of the range of motion (mm) of patient groups with change or no change in disc positions after treatment; related to age, locking duration, maximum mouth opening, lateral and protrusive eccentric movements are seen on Table 2. There was no statistically significant difference between changed and no-changed disc positioned groups related to age, locking duration and change in the disc location (Table 2).

**Table 2:** **Differences in mean values of the range of motion (mm) of patient groups with change or no change in disc positions after treatment; related to age, locking duration, maximum mouth opening, lateral and protrusive eccentric movements**

Variables	Change in disc position (n = 11)	No change in disc position (n = 12)	Overall (n = 23)	t	p-value
Age	25,64 ± 11,01	28,58 ± 10,33	27,17 ± 10,52	0,660	*NS
Locking duration	12,91 ± 10,25	14,5 ± 10,13	13,74 ± 9,99	0,370	*NS
Right/Left lateral movement after treatment	12,33 ± 1,53	11,79 ± 1,28	12,04 ± 1,41	-1,290	*NS
Protrusive movement after treatment	7,64 ± 1,8	7 ± 0,95	7,3 ± 1,43	-1,070	*NS
Maximum mouth opening after treatment	50,73 ± 6,65	47,75 ± 6,03	49,17 ± 6,37	-1,130	*NS

*NS: Not significant.

While the bilateral palpation of the temporal, masseter, sternocleidomastoidmuscles and/or TMJ in patients revealed pain perception prior to treatment; the pain symptoms were eliminated at the end of 24 weeks of treatment in all patients.

Side effects of splint therapy, such as tooth intrusion, tooth loosening or sensitivity on biting, were not present in any of the patients.

## Discussion

Standard treatment protocol for patients with acute ADDWoR mostly starts with manipulation of the mandible to recapture the dislocated disc. If this procedure is successful, an anterior repositioning splint is made. However, when the disc has lost its normal morphology, the chances of maintaining the disc in place become remote [3, 10].

In the authors’ experience, early intervention to treat ADDWoR, yields to good prognosis, particularly in young patients. Thus, it is well worth attempting to reduce the dislocated disc manually several times. However, for chronic cases the prognosis of using these stabilization splints alone has not been predictable, a new treatment protocol using jaw exercises with a pivot splint to mobilize the joint was devised.

A comparative study between jaw-stretch self-exercise and control groups in patients with ADDWoR demonstrated that the exercise group showed significant improvement in both maximum mouth opening and interference with life scores [11]. Another controlled evaluation of non-surgical treatment protocols results suggested that mouth-opening exercise has potential therapeutic effects although gradual reduction of signs and symptoms of ADDWoR was non-specific and was not related to the type of treatment [12]. Also in accordance with the present study results, Haketa et al [13] concluded that, the mouth opening range significantly increased in the exercise group in the 8-week follow-up period.

Any splint may cause an increase in the joint space and stress reduction at articulating surfaces. Stabilization splints have been used in treatment of a large variety of symptoms of TMDs of muscular and/or structural origin [14]. A pivot splint has been thought of having the additional benefit of mobilizing the condyle through the action of jaw-closing muscles over the force vectors created by jaw closing muscles have been found to position the condyle in anterosuperior position, decreasing the stress on the articulating surfaces. Use of elastic bandages from chin to head has been advocated as a mean to apply extraoral forces to cause distraction in the joint [15].

In the present study, exercises were preferred over the use of elastic bandages. These exercises are similar to those recommended in cervical vertebrae problem cases, where soft tissue and joint mobilization is aimed by traction [16]. Traction of the TMJ in a vertical direction may cause an increase in space between bony structures of the joint, creating an environment for the reduction of the dislocated disk and reducing interarticular pressure. Bilateral pivot points at the molar region of splints and extra oral force application provide the desired direction of force. Since constant use of the pivot splint has been associated with intrusion of the teeth under the pivot points, patients in the presented study were instructed to limit the use of the splint to exercise only. With the exception of hygiene, eating and exercise procedures, all patients were asked to wear stabilization splints at all times.

Most patients seek for treatment when pain interferes with daily activities and ADDWoR has been reported to be a painful disorder [17]. Reduction or elimination of pain is an important parameter in evaluation of a therapeutic approach. With similar cases Lundh et al [18] reported a 33%, Okeson et al [19] 50% and Carraro and Cafesse [20] 100% elimination of pain symptoms. However, the Visual Analogue Scale was not used to assess pain as in literature [8]. Pain was determined at the first examination and throughout the follow-up period by bilateral palpation of the TMJ and muscles and asking the patient if they perceived any pain. In our study, clinical assessment at the end of the 24-week treatment period, revealed absence of any joint or muscular pain; thus a 100% success was obtained.

In a study regarding the outcome of arthroscopic surgery, Davis et al [21] reported a mean maximum opening increase of 14.6 mm for unilateral, and 8.9 mm for bilateral ADDWoR cases 6 months after surgery. Eminectomy via open joint surgery has been reported to result in a mean increase of 17.9 mm in maximum opening among 18 closed lock patients [22]. Sodium hyaluronate injection to the superior compartment of the TMJ has resulted in 17.1 mm increase for an ADDWoR cases [23]. Dimitroulis [24] reported an increase in mean maximum opening from 24.6 mm to 42.3 mm when closed lock cases were treated with arthrosynthesis and lavage followed by manipulation to reduce the discs. Murakami et al [25] compared the outcome of arthrosynthesis, arthroscopy and nonsurgical treatment approaches for ADDWoR cases. The nonsurgical treatment consisted of non-steroidal anti-inflammatory drugs and muscle relaxants for the first two weeks, followed by manipulation to reduce the discs. Pivot splints were used for up to 12 weeks for cases that showed no improvement in this group. When success criteria were defined as absence or significant reduction of pain maximum opening beyond 38 mm and 6 mm minimum lateral and protrusive movements, 55.6% of the nonsurgical group, 70% of the arthrosynthesis and 91% of the arthroscopic surgery groups were found to be successful.

Another approach to evaluate the outcome of TMD treatment involves measurement of mandibular range of motion including not only maximum interincisal opening but left and right lateral and protrusive movements as well. 40–58 mm has been reported as average amount of maximum interincisal opening and 8–10 mm as lateral and protrusive movements [26]. In the presented study, significant increase of 20.43 mm in maximum opening, 4.43 mm in right/left lateral movement and 3.21 mm in protrusive movements were observed, when comparing the baseline measurements with post-treatment outcomes. These results are well within the normal range reported by previous studies [21–24]. The results also indicated that the most dramatic improvement in the range of motion was within the first 4 weeks of treatment. During the treatment, the progress of improvement gradually decreased but continued until the twenty forth week when the study was concluded.

Choi et al. [27] reported that, conservative treatment procedures that were used at ADDWoR cases were beneficial not because they change the position of displaced disk, instead they increase the mobility of condyle and an adaptation of posterior attachments occurs. Kirk [28] reported that the clinical success of treatment did not mean a change of anatomic relationships of TMJ. McNeill [29] reported that without recapturing the displaced disk, normal function could be obtained by the adaptation of retrodiscal tissue. The results of the presented study agree with previous studies.

This study have not compared the efficacy of pivot splint with any other treatment procedure, however, the symptoms of TMD have improved after 24-week treatment protocol. Since normal mandibular range of motion was reestablished and pain was absent after treatment among all patients, the treatment concept investigated seemed effective for cases diagnosed as having ADDWoR.
